# Acute postoperative pain after arthroscopic rotator cuff surgery: A review of methods of pain assessment

**DOI:** 10.1051/sicotj/2018042

**Published:** 2018-11-22

**Authors:** Jacob Korsbæk Rasmussen, Lone Nikolajsen, Karen Toftdahl Bjørnholdt

**Affiliations:** 1 Aarhus University, Vennelyst Boulevard 4, Aarhus Denmark; 2 Department of Anesthesiology and Intensive Care, Aarhus University Hospital, Research, C319, Palle Juul Jensens Boulevard 99, Aarhus Denmark; 3 Department of Orthopedic Surgery, Horsens Regional Hospital Sundvej 30 Horsens Denmark

**Keywords:** Rotator cuff surgery, Postoperative pain, Pain intensity, Pain rating scale, Analgesic consumption.

## Abstract

*Introduction:* Pain can be severe during the first days after arthroscopic surgery, and acute pain is an important outcome in clinical trials of surgical technique or anaesthetic strategy. A standardized, validated method of assessing acute postoperative pain would improve the quality of clinical studies, and facilitate systematic reviews and meta-analyses. A step on the way towards this standard is to investigate the methods most commonly used in recent literature.

*Methods*: PubMed and CINAHL databases were searched, including studies of arthroscopic rotator cuff surgery with a primary pain-related outcome during the first postoperative week, published in English from 2012 to 2017.

*Results*: A total of 47 studies were included, all measuring pain intensity using a pain rating scale. Most frequently used was the visual analogue scale using the anchors “no pain” and “worst pain imaginable”, with recordings at 1, 2, 4, 6, 8, 12, and 24 hours postoperatively. A total of 34 studies recorded analgesic consumption, usually as average cumulated consumption in mg. Time to first analgesic request or first pain were recorded in 11 studies, and 4 different starting points were used.

*Discussion*: This review describes the currently most common methods of assessing acute postoperative pain in clinical trials of arthroscopic shoulder surgery involving rotator cuff repair, and the large variety of methods applied. Based on this study and international guidelines, several recommendations on how to measure and report postoperative pain outcomes in future trials are proposed.

## Introduction

Pain assessment is fundamental in orthopedic surgery to evaluate disease severity as well as postoperative improvement. When evaluating new surgical interventions and analgesic methods, clinical trials often use acute postoperative pain as an outcome. Recent quality improvement guidelines of acute postoperative pain from the American Pain Society [1], a review [2] and a study protocol [3] of acute postoperative pain after shoulder surgery have defined outcomes to be included in postoperative pain trials, such as “pain intensity” [4,5]. However, recommendations for outcome measurements and recordings are lacking [6]. For example, pain intensity may be reported through various pain rating scales. As a result, trials have assessed postoperative pain in a wide variety of ways [2,7,8].

A standardized, validated method of assessing acute postoperative pain would improve the quality of clinical studies and facilitate future systematic reviews and meta-analyses. Thus, the aim of this study was to investigate the methods used in recent literature, focusing on ratings of pain intensity, time points and number of measurements, analgesic consumption, and time to first analgesic request or first pain.

## Materials and methods

### Data sources and searches

The databases PubMed and CINAHL were used to identify studies on shoulder surgery and postoperative pain. Subject-specific terms relating to any type of shoulder surgery were identified in the MeSH tree, PubMed, and in Headings, CINAHL. Any subject-specific terms unavailable in the indexed headings were searched as free text in title and abstract, such as subacromial decompression. All relevant MeSH terms/headings not exclusive to surgery or pathology of the shoulder, such as arthroscopy or bursitis, were combined with a shoulder-related MeSH term/heading. For MeSH terms introduced in 2012 or later, the previous MeSH term indexing was added to the search. To include articles with incomplete indexing processes in PubMed, a free text search in title and abstract was conducted for the last 2 years, using the subject-specific terms.

### Selection of included studies

The identified articles from the systematic literature search were imported to Covidence, a web-based systematic review software (Veritas Health Innovation, Melbourne, Australia) [9]. Two reviewers, JKR and KTB, assessed the articles individually, and consensus was sought through discussion in case of disagreement. The inclusion criteria were: shoulder surgery, report of any kind of pain assessment within the first postoperative week, and published in English from 30 June 2012 to 30 June 2017. As this search provided more relevant studies for inclusion than first anticipated, this review focused on arthroscopic shoulder surgery including rotator cuff surgery, and pain as the primary outcome. If no primary outcome was specified, the first outcome mentioned in the study's material and methods section was considered primary. Finally, the bibliographies of the included studies and related review articles were manually reviewed for other relevant studies, which were then included if they met the inclusion criteria.

### Data collection

From the included studies, the following data was recorded: first author, year of publication, study design, surgical procedure, intervention, total number of patients, primary outcomes, and pain-related outcomes. Details regarding the methods of pain assessment were recorded as follows: data collection by staff (telephone or personal interviews) or by patients themselves (pain diaries or questionnaires), pain scale used, phrasing of pain anchors, measurement at rest or during activity, measurement of present/average/worst pain, time-points of measurements on postoperative day (POD) 1 and number of measurements daily until POD7, analgesic consumption, time to first analgesic request or first pain.

## Results

**Figure 1:**
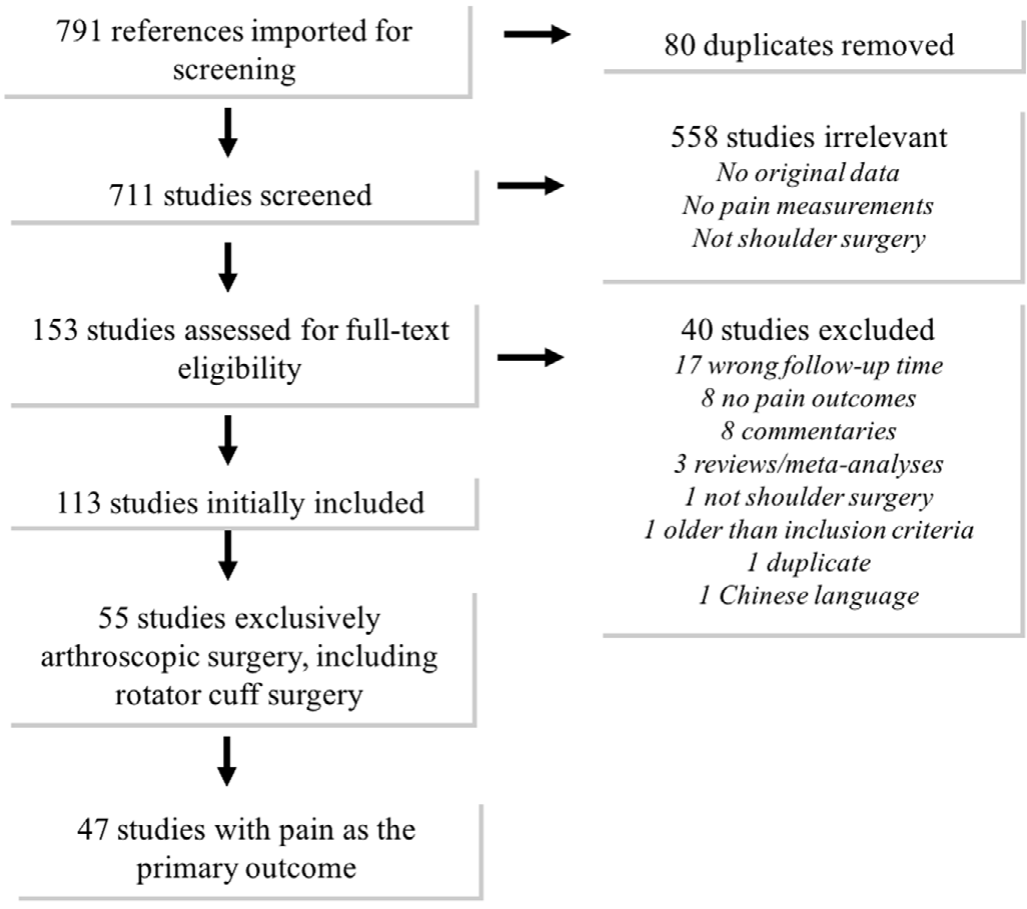
Flowchart.

A total of 711 non-duplicate articles eligible for screening of title and abstract were identified, of which 153 were further assessed in full text; 47 studies met the inclusion criteria (Figure 1). The 47 included studies included randomized controlled trials (RCTs) (n = 33), non-randomized comparative studies (n = 6), quasi-RCT (n = 1), cohort studies (n = 4), case series (n = 2), and one up-and-down dose finding study (Tab. 1). The study populations were patients undergoing arthroscopic rotator cuff surgery exclusively (n = 29) or in combination with other arthroscopic shoulder surgery (n = 18). The number of patients analyzed in the studies ranged from 24 to 1624 (mean 122). Interventions (Tab. 1) were performed by orthopedic surgeons, anesthesiologists, physiotherapists, nurses, and industry pharmacists.

**Table 1:** The 47 included studies containing arthroscopic rotator cuff surgery with pain as the primary outcome.

			Pain-related outcomes		
Author, year	Study design	Intervention	Pain scale	Analgesic consumption	Time to first request
Abdallah et al., 2016	RCT	ISB + dexmedetomidine adjuvant vs. IV vs. none	VAS	+	+
Ahn et al., 2016	RCT	Pregabalin vs. placebo before surgery	NRS	+	–
Aksu et al., 2015	RCT	ISB vs. intraarticular bupivacaine vs. none	VAS	+	+
Alemanno et al., 2014	Non-randomized	MIB levobupivacaine vs. levobupivacaine + buprenorphine vs. levobupivacaine + tramadol	VAS	–	+
Alemanno et al., 2016	Non-randomized	MIB levobupivacaine vs. levobupivacaine + thiamine	VAS	–	+
Alfuth et al., 2016	RCT	Cold compression vs. cold pack	VAS	–	–
Basat et al., 2016	Case-series	Suprascapular and axillary nerve block	VAS	–	–
Cheng et al., 2016	Cohort	Correlation of fibromyalgia and postoperative pain	10-point scale	+	+
Cho et al., 2015 Feb	Non-randomized	ISB vs. none	VAS/Faces*	+	–
Cho et al., 2015 May	Non-randomized	Zolpidem vs. none	VAS	+	–
Choi et al., 2015	RCT	Stellate ganglion block vs. none	VAS	+	–
Cuff et al., 2016	Cohort	Correlation of preoperative factors and postoperative pain	VAS	–	–
D'Ambrosi et al., 2016	RCT	Platelet rich plasma during surgery vs. none	VAS	–	–
Desmet et al., 2015	RCT	dexamethasone IV vs. placebo	Likert	+	+
Dhir et al., 2016	RCT	Suprascapular and axillary nerve block vs. ISB	NRS	+	–
Erden et al., 2017	quasi-RCT	Standard pain assessment protocol vs. routine pain assessment	VAS/NRS	+	–
Faria-Silva et al., 2016	RCT	Block with clonidine vs. block without clonidine	NRS	+	–
Han et al., 2013	RCT	Multimodal local injection vs. i.v. patient-controlled analgesia	VAS	+	–
Jo et al., 2014	RCT	Multimodal local injection vs. placebo	VAS	+	–
Khashan et al., 2016	RCT	Preincisional intraarticular morphine vs. ketamine + morphine vs. placebo	NRS	+	–
Kim et al., 2016	RCT	ISB 0.2% ropivacaine vs. ISB 0.75% ropivacaine vs. cervical epidural block	VAS	+	–
Kraeutler et al., 2015	RCT	Compressive cryotherapy vs. ice	VAS	+	–
Lane et al., 2014	case series	Same day discharge after GA and ISB	NRS	–	–
Lee et al., 2015	RCT	Local anesthetic injection in the GH vs. the SA vs. both	VAS	+	–
Lee et al., 2014	RCT	SSNB + ANB vs. SSNB + placebo	VAS	–	–
Lee et al., 2015	RCT	Arthroscopy guided SSNB vs. placebo	VAS	+	–
Lee et al., 2012	Non-randomized	ISB vs. SSNB + ANB vs. none	VAS	+	–
Lehmann et al., 2015	RCT	GA vs. GA + ISB vs. ISB	NRS	+	+
Merivirta et al., 2013	RCT	Subacromial bupivacaine infusion vs. transdermal fentanyl patch	NRS	+	–
Merolla et al., 2015	RCT	Dietary supplement vs. placebo	VAS	+	–
Park et al., 2016	RCT	SSNB + ANB vs. SSNB vs. none	VAS	–	–
Perdreau et al., 2015	RCT	Multimodal local injection vs. placebo	VAS	+	+
Rubenis et al., 2015	Non-randomized	Undersurface rotator cuff repair vs. bursal-side rotator cuff repair	Likert	–	–
Ryu et al., 2015	RCT	Supraclavicular brachial plexus block vs. ISB	NRS	+	+
Salviz et al., 2013	RCT	ISB vs. ISC vs. GA	NRS	+	+
Saritas et al., 2015	RCT	Intraarticular magnesium sulphate vs. placebo	VAS	+	–
Schwartzberg et al., 2013	RCT	Subacromial bupivacaine infusion vs. placebo vs. none	VAS**	+	–
Shin et al., 2014	RCT	ISC infusion + PCA vs. ISC PCA vs. IV PCA	NRS	+	–
Shin et al., 2016	RCT	C5-approach ISB vs. ISB vs. none	NRS	–	–
Tham et al., 2013	Cohort	Correlation between tendon thickness and pain	Likert	–	–
Vorsanger et al., 2013	RCT	Tapentadol vs. oxycodone	NRS	+	–
Wei et al., 2014	Up-and-down	Infusion rate of ropivacaine in ISC	NRS	+	–
Wiegel et al., 2017	RCT	SSNB vs. ISB	NRS	+	–
Woo et al., 2014	RCT	Ketamine infusion during GA combined with single-shot ISB	NRS	+	+
Woo et al., 2015	RCT	Dexamethasone on the duration of single shot ISB	NRS	+	+
Yeo et al., 2017	Cohort	Correlation of rotator cuff tear area and postoperative pain	Likert	–	–
Yun et al., 2012	RCT	Patient-controlled analgesia vs. intravenous patient-controlled analgesia	VAS	+	–

ANB, axillary nerve block; GA, general anesthesia; ISB, interscalene brachial plexus block; ISC, interscalene catheter; MIB, middle interscalene block; NRS, numeric rating scale; PCA, patient controlled analgesia; RCT, randomized controlled trial; VAS, visual analogue scale; SSNB, suprascapular nerve block.

* Cho et al. report using VAS but report that participants had to “point to the position on the line between the faces”.

** Schwartszberg et al. report using a VAS, but describe pain scores being obtained verbally.

### Primary outcome

Pain intensity was measured using a rating scale in all 47 studies (Tab. 1), and was the primary outcome in 37 studies. Analgesic consumption was reported in 34 studies and was the primary outcome in 6 studies [10–15]. The time to first pain or analgesic request was reported in 11 studies and was the primary outcome in 5 studies [10,16–19] (one of which also had analgesic consumption as a primary outcome [10]). In 8 studies, a rescue analgesic was given based on pain scale ratings [10–12,15,17,20–22].

### Methods for data collection

Staff-administered pain questionnaires were applied in 27 out of 47 included studies [11–14,16–21,23–39]. Nine studies [10,18,39–45] used a patient-administered pain questionnaire, and 12 studies [15,46–56] did not specify their method. One study used a staff-assessed method in the hospital and a self-assessed method after patient discharge [18].

### Ratings of pain intensity

Of the 47 studies, 26 used the visual analog scale (VAS), 17 used the numeric rating scale (NRS), 4 used a Likert scale, and a single study used a combined VAS and Faces Pain Scale (Tab. 1).

**Figure 2:**
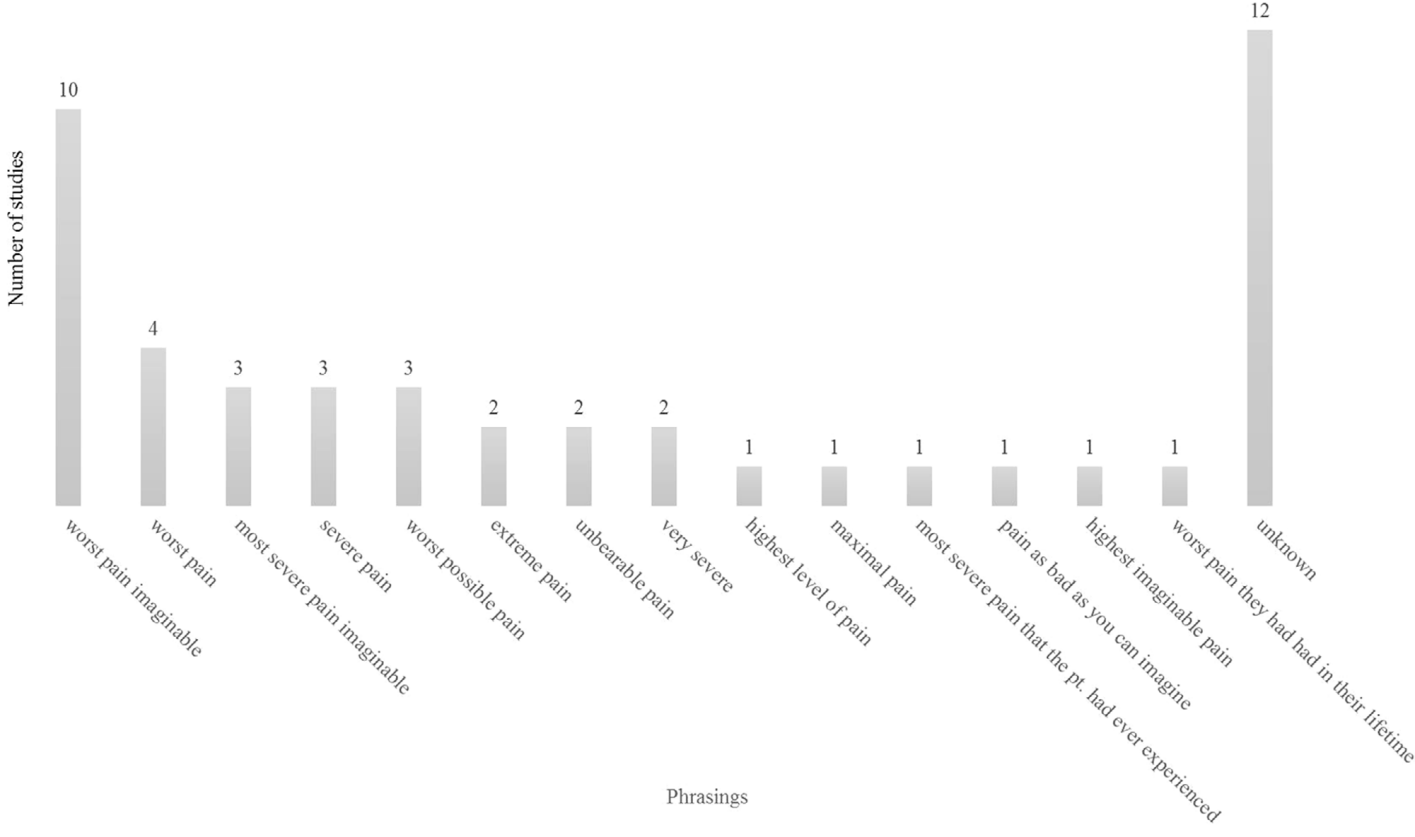
Maximum pain level anchors used n = 47 studies. “Worst imaginable pain” was grouped with “worst pain imaginable”.

The VAS, NRS, and Likert scale all used similar minimum anchors such as “no pain”. To describe higher pain levels, the scales used 14 different anchors in total (Figure 2) with “worst pain imaginable” [11,12,15,26,36,38,43,48,51,54] being the preferred anchor. The second most frequent anchor was “worst pain” [10,28,33,34] and then successively “most severe pain imaginable” [19,20,37], “severe pain” [18,32,46], and “worst possible pain” [29,41,47]. Twelve studies did not report the used anchors [13,16,17,27,31,35,40,49,50,55–57].

Pain ratings were specified to be at rest or during activity in 11 studies [10,13,16,23,26–28,32,44,55,58], of which 3 studies [10,16,26] specified pain to be exclusively measured at rest. Eight out of the 11 studies described pain during activity and the definitions were: movement during cough and mobilization [27], motion attempts [28], shoulder movements [58] during activities such as dressing or during transfer from lying to sitting [23], passive motion [55], during overhead activities and sleep [44]; two studies did not specify the activity [13,32]. The remaining 36 studies did not specify whether pain was measured during activity or at rest.

The following 6 studies asked patients to rate pain as either present pain, least pain, worst pain, or average pain during a specified period of time, namely average pain score for the day [25], daily average pain [40], overnight pain [29], level of pain during sleep [45], overall pain [32], and average of least and worst pain [13]. The remaining studies did not report these details of the pain recordings.

### Number of measurements and time points

**Figure 3:**
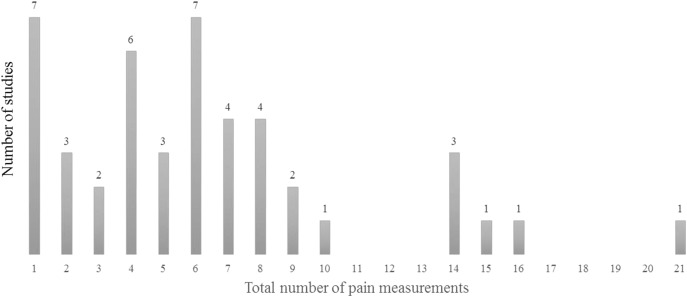
Total number of pain recordings during the first postoperative week n = 45 studies.

The total number of pain recordings within the first seven PODs was extracted from 45 studies (Figure 3); two studies did not report the total number of recordings [15,54]. The mean number of recordings within the first postoperative week was 6.3 (0–21).

**Figure 4:**
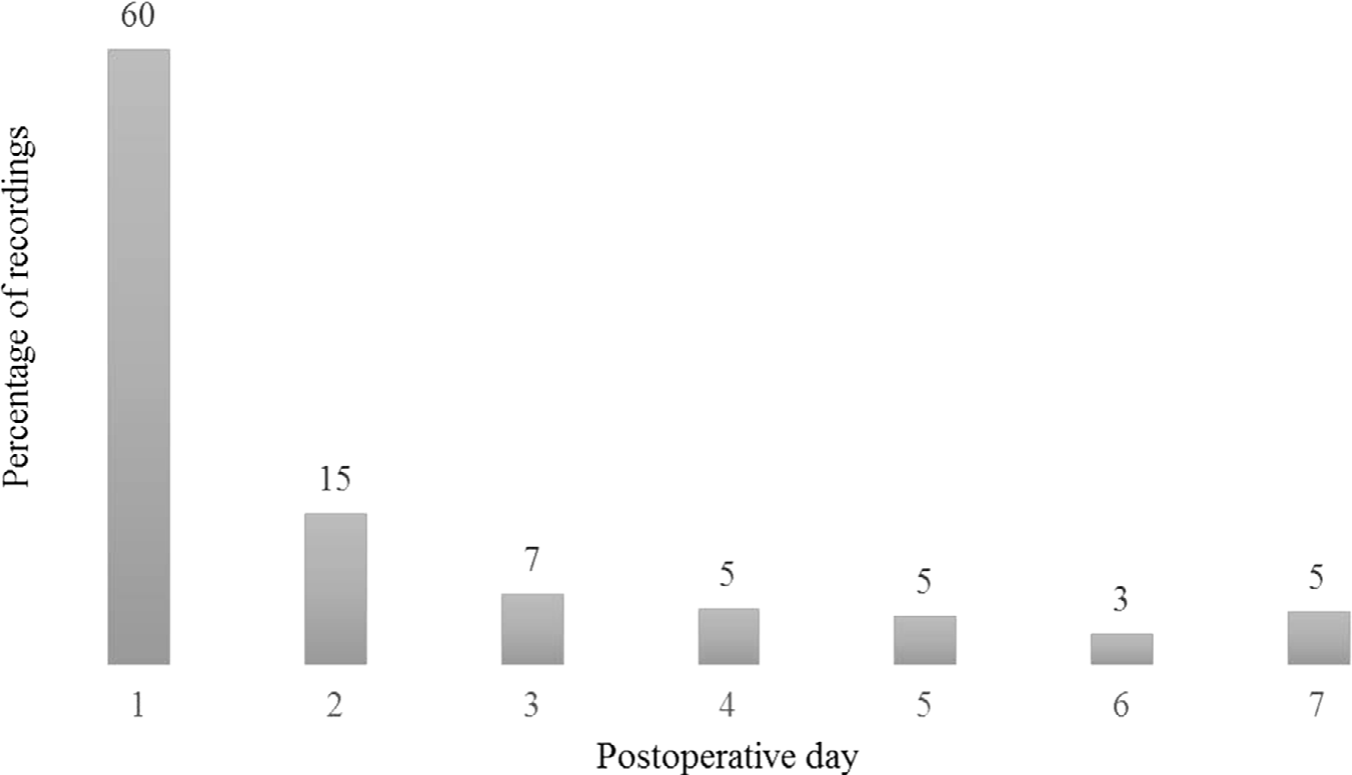
Percentage of pain recordings within the first seven postoperative days. The data represent 268 recordings, by any applied pain rating scale, extracted from 45 studies.

**Figure 5:**
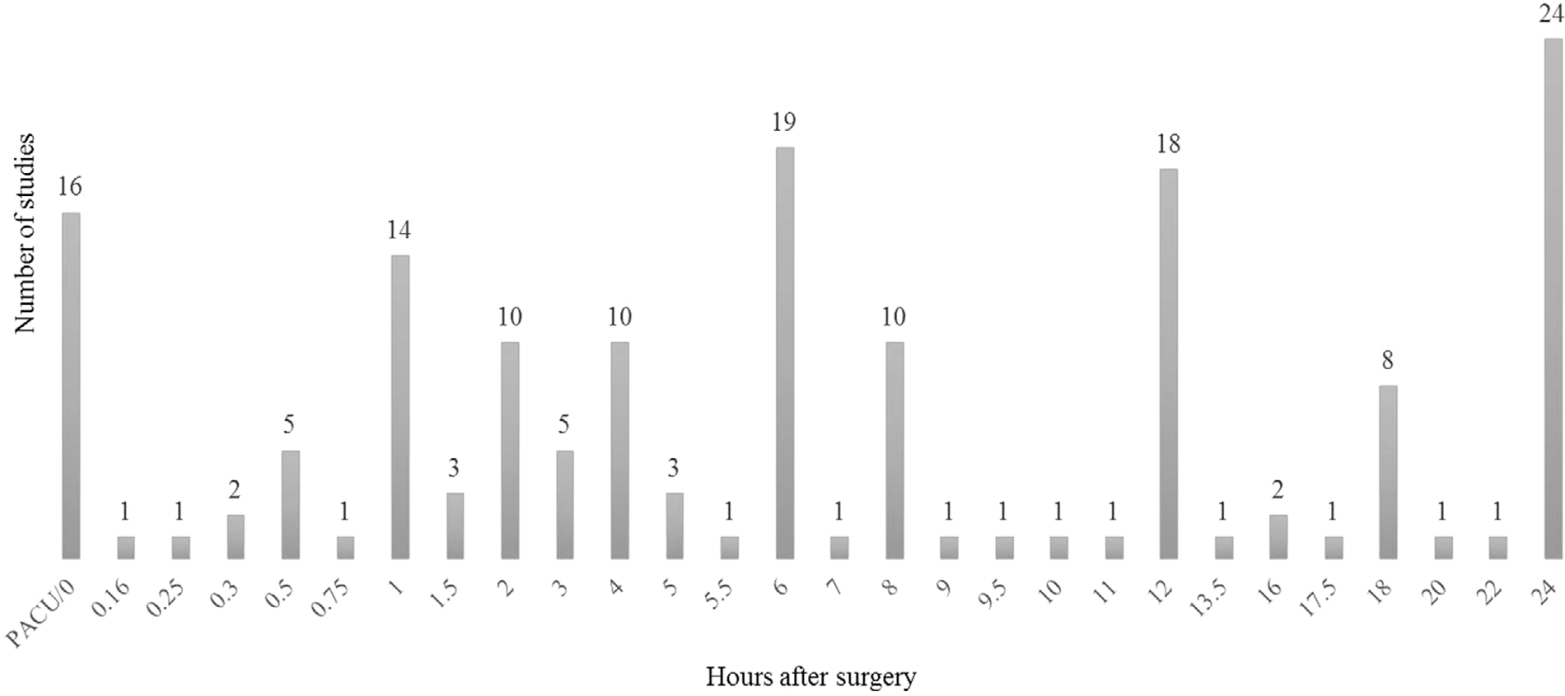
Number of studies using a specific time point, first 24-hour postoperative pain recordings. Data from 32 studies are included with a total of 162 measurements, equaling a mean of 5.1 pain recordings per study on POD1. *Erden et al. measured pain within time intervals 0–3, 4–7, 8–11, 12–15, 16–19, 20–24 hours. To include the data, the middle range was chosen as time of measurement. **Jo et al. recorded pain 5 hours postoperatively and then three times daily (at 0:00 am, 9:00 am, and 5:00 pm). Since we do not know what time of day the patients were operated, only the first recording after 5 hours is included in the graph. ***Ryu et al. measured pain hourly until request of rescue analgesic which was requested at a mean of 11 hours and 45 minutes postoperatively on which basis we added these 11 pain recordings to the graph.

The distribution of recordings during the first seven PODs could be extracted from 45 studies (Figure 4). The majority of pain recordings were made on POD1, totaling 60% of cumulated number of recordings within the first postoperative week, equivalent to a mean of 5.1 (0–11) recordings. The number of pain recordings decreased from POD2 to POD6 with a small increase in POD7. The time points in POD1 were specified in 32 of the included studies and represented 28 different time points (Figure 5) [10–12,14,19–21,23,26–28,30,31,33–38,43,46–52,54–57,59]. The most frequently used time points were: post-anaesthesia care unit, 1, 2, 4, 6, 8, 12, and 24 hours postoperatively.

### Analgesic consumption

In the 34 studies reporting analgesic consumption, different methods were used. Some studies used more than one method due to variations in analgesic protocols, such as different scheduled analgesics and rescue analgesics. In most cases, only the rescue analgesics were recorded (in mg or number of doses) [14,21,24,47,49], or the number of patients requesting [34,54,56] (or not requesting) analgesics [19]. For patients using patient-controlled analgesia (PCA, usually with intravenous opioid), the number of attempts, number of doses, and/or cumulated dose (for example during 24 hours) were reported [31,37,41,48,50,52,57]. Some studies reported total consumption of morphine/opioids [12,13,47] or cumulated amount of all analgesics [10,15] during a specific period of time.

### Time to first analgesic request or first pain

This was the least used primary pain outcome. Studies reporting this outcome were mainly, but not limited to, trials of peripheral nerve blocks. Four studies used time to first pain [10,13,15,34], and seven studies used time to first request of analgesics [12,16–19,37,52]. Different starting points were used when reporting time to first analgesic request or first pain: time from injection of the local anesthetic solution [16,18], time from extubation [52], time from closure [15], and time from arrival at the post-anesthesia care unit [37].

## Discussion

When screening the 711 abstracts for the current study, many of the first discarded 558 studies were found to use questionnaires with joint-specific or health-related quality of life composite scores (such as DASH score, American Shoulder and Elbow Surgeons (ASES) score, Oxford shoulder score, and Western Ontario Rotator Cuff Index). Since data on pain assessment could not be extracted from these composite scores, these studies were unsuitable for further analysis of postoperative pain. Currently this issue is being investigated by others, for example Gagnier et al. [3] who has published a study protocol with the purpose of creating a core outcome set for clinical trials of people with shoulder pain as the lack of uniformity in outcome measures across clinical trials limits interstudy comparison and the ability to pool data for meta-analyses.

The majority of included studies used the VAS to measure pain intensity. The literature generally indicates that the VAS and the NRS are interchangeable, and some studies report coherence between the VAS and NRS pain rating scales [4,60,61]; others found no coherence at specific pain levels [62]. On basis of international guidelines, literature recommendations [1,62,63,65], and practical applications where the NRS is seemingly easier to administer than the VAS [63,64] (since it requires no remedies), this study recommends using the NRS to measure pain intensity.

Though the majority of included studies reported the chosen pain rating scale, such as the VAS or NRS, a description of scale use is rare to find, thus obscuring whether the scales were used as originally intended. In at least two of the included studies, the actual use of the VAS was described as a Faces Pain Scale and the NRS, respectively [24,35].

The pain rating scales used 14 different anchors when phrasing maximum pain levels. Although there was little variation in the phrasing, the lack of consistency is noticeable. Hawker et al.'s [63] review article from 2011 reported that the VAS was designed using the anchor “severe pain”, whereas the NRS was designed with the anchors “pain as bad as you can imagine” and “worst pain imaginable.” Being able to compare future studies, it is important they apply the same scale and anchors, i.e., “no pain” and “worst pain imaginable.”

Few studies reported if pain was measured during activity or at rest. Recording pain during activity increases sensitivity due to higher pain scores, and reflects patients' functional levels. Based on international recommendations [1,65], it is recommended to report both pain at rest and during activity. Focus is thus drawn on pain during deep breathing and coughing to reduce risks of cardiopulmonary and thromboembolic complications after surgery [4].

The majority of pain recordings within the first postoperative week were recorded on POD1. When looking at the time points on POD1, 28 different time points were used in 32 studies. Naturally, some of the variation is a result of peaks of interest, reflecting different interventions. However, some standardization seems possible. It is thus recommended not to report “post-anesthesia care unit” as a time point but rather to use the most common time points of 1, 2, 4, 6, 8, 12, and 24 hours, preferably starting from injection of local anesthetic or from extubation, dependent on the intervention investigated. Too many measurements will cause problems if not using the proper statistical methods, but this is beyond the scope of this review.

It is important to include the outcome of analgesic consumption, as the pain intensity ratings do not necessarily reflect the patients' wishes for additional opioids [66,67]. Some studies showed discrepancies between pain levels and analgesic consumption, with one outcome being statistically significant and the other not [10,11,13,18,21,41,48,50,52]. It is therefore advisable to report both analgesic intake as well as pain intensity ratings [68].

Reporting morphine equivalent doses, as was done in some studies, bears the risk of error in the conversion factors, but may be necessary if the protocol involves more than one opioid and facilitates interstudy comparison.

Time to first analgesic request or first pain is related to the “time to remedication,” often used in studies with nerve blocks, illustrating when patients will need further attention [5]. Time to first pain would be more sensitive, as onset of pain may precede the first analgesic request, especially in patients reluctant to consume medication. It is important to use the recommended starting points here as well, such as time from injection or extubation.

### Limitations

Despite a wide-ranging search strategy, the risk of missing relevant studies cannot be excluded. However, the considerable variation regarding outcomes and reported methods indicates that a comprehensive search was conducted. Also, two databases were searched for relevant literature, thus strengthening the study. It is assumed that the findings from the current study can be generalized to other populations with acute postoperative pain as there is no apparent reason to assume that postoperative pain following rotator cuff surgery is measured differently from other types of surgery, but this may be a subject for further investigations.

## Conclusion

A large variety in the reporting of postoperative pain outcomes in the 47 included studies was shown. Based on this study and international guidelines, future postoperative pain trials are recommended to include the following outcomes: pain intensity using the NRS with the anchors “no pain” and “worst pain imaginable,” recorded during activity and at rest at 1, 2, 4, 6, 8, 12, and 24 hours postoperatively. Analgesic consumption should also be included and reported as cumulated rescue analgesic medication in mg, if possible converted to morphine equivalents.

Assuming that postoperative pain following rotator cuff surgery is not measured differently from other types of surgery, the applications of this study may be expanded to clinical trials involving patients undergoing other surgical procedures.

### Conflict of interest

The authors have not received grant support or research funding and do not have any proprietary interests in the materials described in the article.
